# Wheelchair-Mounted Upper Limb Robotic Exoskeleton with Adaptive Controller for Activities of Daily Living

**DOI:** 10.3390/s21175738

**Published:** 2021-08-26

**Authors:** Bridget Schabron, Jaydip Desai, Yimesker Yihun

**Affiliations:** 1 Neuro-Robotics Lab, Biomedical Engineering, Wichita State University, Wichita, KS 67260, USA; bcschabron@shockers.wichita.edu; 2 Robotics and Control Lab, Mechanical Engineering, Wichita State University, Wichita, KS 67260, USA; yimesker.yihun@wichita.edu

**Keywords:** electromyography, artificial neural network, exoskeleton, assistive technology, robotics, hand gestures

## Abstract

Neuro-muscular disorders and diseases such as cerebral palsy and Duchenne Muscular Dystrophy can severely limit a person’s ability to perform activities of daily living (ADL). Exoskeletons can provide an active or passive support solution to assist these groups of people to perform ADL. This study presents an artificial neural network-trained adaptive controller mechanism that uses surface electromyography (sEMG) signals from the human forearm to detect hand gestures and navigate an in-house-built wheelchair-mounted upper limb robotic exoskeleton based on the user’s intent while ensuring safety. To achieve the desired position of the exoskeleton based on human intent, 10 hand gestures were recorded from 8 participants without upper limb movement disabilities. Participants were tasked to perform water bottle pick and place activities while using the exoskeleton, and sEMG signals were collected from the forearm and processed through root mean square, median filter, and mean feature extractors prior to training a scaled conjugate gradient backpropagation artificial neural network. The trained network achieved an average of more than 93% accuracy, while all 8 participants who did not have any prior experience of using an exoskeleton were successfully able to perform the task in less than 20 s using the proposed artificial neural network-trained adaptive controller mechanism. These results are significant and promising thus could be tested on people with muscular dystrophy and neuro-degenerative diseases.

## 1. Introduction

Actively-actuated exoskeletons with joints with several degrees-of-freedom (DOF) are complex and powerful due to the use of actuators for the movement of the joint compared with both passively and non-actuated exoskeletons. The use of active actuators enhances the ability to move multiple joints at the same time, creating a greater work space to perform ADL [1] and also offering greater precision compared with the gravity-offsetting systems of springs, elastic bands, and counter-weights used in passive and non-actuated exoskeletons. Currently, there are several actively-actuated exoskeletons on the market, including the Orthojacket [2], Multimodal Neuroprosthesis for Daily Upper Limb Support (MUNDUS) [3], the robotic upper-extremity repetitive trainer (RUPERT) [4], the biomimetic orthosis for the neurorehabilitation of the elbow and shoulder (BONES) [5], and the motorized upper-limb orthotic system (MULOS) [6]. Most of these actively controlled exoskeletons require a precise control and user interface system that infers a user’s input to control the actuators in real-time. There are a wide variety of options for intuiting user input; the simplest and most frequently used option is a switch, key, button, or joystick [7,8]. In fact, a survey of upper limb hybrid exoskeletons in 2017 noted that these were utilized in 50% of hybrid exoskeletons (exoskeletons with functional electrical stimulation) [8]. The signal from these kinds of sensors is easier to receive and most directly interpret. However, this also limits user movement, taking away the user’s ability to perform activities with both arms while also controlling the exoskeleton [8]. Another method is the use of voice commands, which free up the user’s hands but are difficult to utilize in crowded or noisy environments. There is also a visual system that monitors eye movement to infer user intent, which is limiting due to the placement of the eye-tracking unit being restricted to a table [8].

Biological signals are also used as an input to drive the exoskeletons, as well as for more general feedback to gather information on fatigue or the muscle groups utilized during use of the exoskeleton. Electroencephalography (EEG) and surface electromyography (sEMG) are the most complex inputs used for deriving user intent to control exoskeletons [8]. EEG, or electrical signals of the brain, are occasionally used for brain–machine type interfaces for exoskeleton control [8]. However, when utilized to control exoskeletons, the EEG signals are not intuitive and are often used with predefined trajectories, such as for the BCI-Controlled Wearable Robot in 2014 and the BCI-Controlled Exoskeleton in 2015, or predefined trajectories in addition to a joystick for the degree of movement for the THINK2GRASP exoskeleton in 2013 [8]. The MUNDUS is another example that uses a brain–computer interface (BCI), but it combines EMG with a USB button and eye-tracking user input options as well [3].

The use of sEMG, or surface-level electrical muscle signals, is a less complicated input than the brain but nonetheless can be quite complex. Some exoskeleton controllers have used simple thresholding on EMG signals [9]. Others take more complex approaches, offering more possibilities for using the signals, such as a neurofuzzy modifier to estimate joint torques from EMG signals [10,11]. While this concept of joint torque estimation is promising, it also requires a higher number of sensors, with 16 wired sEMG sensors along deltoid, pectoral, biceps, triceps, flexor, and extensor muscles of the arm in one study [10] and 14 wired sEMG signals covering many of the same muscles in another study [11]. Although these studies have shown the promise of such a system, commercial applicability would be difficult to attain given the number and wide-ranging placements of sensors; imagine a patient trying to put even 14 sensors on alone, or having to them put on his/her arms and shoulders, each and every day. Of course, there is the possibility of the development of a sleeve and electrodes that do not require re-placement every day, but the complexity from the sheer number of signals still remains.

Another study estimated the force of antagonist and agonist muscles of the arm (biceps—short and long head and flexor and extensor carpi radialis) using the Hill model and sEMG signal inputs from those muscles and the use of different forces as an input for a proportional–integral (PI) controller. The same study also looked at a different control method that used the same sEMG signals but instead utilized machine learning, specifically linear discriminant analysis, to classify movements [12]. A study using the ETS-MARSE related sEMG signals to muscle forces and torques using a proportional constant for each muscle [13]. These various studies show the interest and promise of EMG, as well as the limitations of the current use of EMG input. This research article proposes a compact, additive-manufactured, and actively-actuated 3 DOF wheelchair-mounted upper limb robotic exoskeleton that offers shoulder horizontal abduction and adduction, shoulder flexion and extension, and elbow flexion and extension movements based on artificial neural network-trained hand gesture recognition and an adaptive controller mechanism to ensure the user’s safety while maneuvering the assistive exoskeleton for daily activities. Eight able-bodied participants without upper limb movement disabilities were recruited in this feasibility study to evaluate the response of the proposed system during individual joint control movements followed by a water bottle pick and place task using hand gestures. Quantitative data sets and subjective feedback were acquired to evaluate the proposed technology, which has the potential to be utilized in the near future on people with muscular dystrophy and neuro-degenerative diseases that limit a person’s ability to move their arms in three-dimensional space.

## 2. Methods

The proposed artificial neural network-trained hand gesture recognition system based on sEMG and an adaptive controller mechanism was implemented on an in-house-built wheelchair-mounted upper limb robotic exoskeleton. The exoskeleton was intended to be used for both assistive and rehabilitative tasks for people with upper-limb impairment. The mechanism was designed based on human anatomical data and the desired workspace to accommodate functional ranges of motions to perform some ADLs. The system is equipped with safety features of mechanical joint limits and electronic sensors to communicate with the user through physiological data, such as EMG and force myography signals (FMG).

### 2.1. Wheelchair-Mounted Upper Limb Robotic Exoskeleton Design

Human anatomical data from studies on human anatomy were collected to create the computer-aided design (CAD) model to locate the joints and allow flexion/extension and abduction/adduction at the shoulder joint and flexion/extension for the elbow. To accommodate ranges of sizes of subjects while minimizing stresses at the joints, the design incorporated adjustable linkages. The computer-aided models of the wheelchair-mounted robotic exoskeleton were created using SolidWorks ^®^. The entire prototype was divided into three assemblies, named (1) the adjustable elbow forearm assembly, (2) the shoulder flexion/extension assembly, and (3) the shoulder horizontal abduction/adduction assembly.

**Figure 1:**
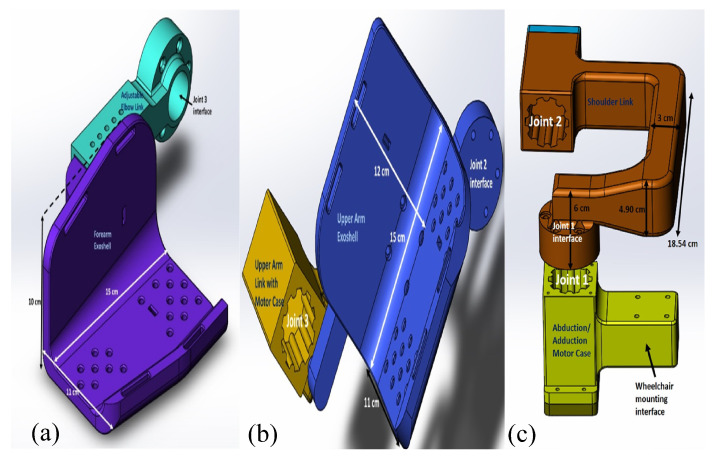
(**a**) Adjustable elbow forearm assembly, (**b**) shoulder flexion/extension assembly, and (**c**) shoulder horizontal abduction/adduction assembly of the exoskeleton.

The adjustable elbow forearm assembly of the prototype consists of a forearm exoshell part and an adjustable elbow link. The adjustable link allows the total length of the lower arm assembly to vary from 21.4 to 26.4 cm, depending on the user’s arm length. The forearm exoshell is an open L-shaped piece and is rounded at the corner edges in order to better fit the user, with the strap channels following the rounded contours. At the interface, filleted holes are added for ventilation to prevent the arm from overheating. The assembly of the forearm shell (purple) and the adjustable link (cyan) is shown in Figure 1a.

An upper arm exoshell attaches to an upper arm link with a motor case to create the shoulder flexion/extension assembly, as shown in Figure 1b. Similar to the forearm exoshell, the upper arm exoshell has holes for ventilation and rectangular channels for straps. One link on the side of the upper arm exoshell helps to keep alignment with elbow joint. The last main assembly is the shoulder horizontal abduction/adduction assembly, as shown in Figure 1c.

**Figure 2:**
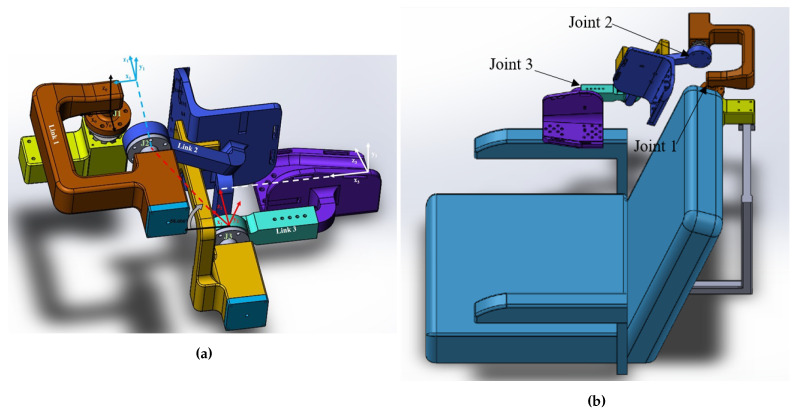
(**a**) Assembly of the exoskeleton, (**b**) assembly of the exoskeleton mounted on the wheelchair.

The individual parts were assembled as shown in Figure 2a and mounted on a wheelchair, as shown in Figure 2b. The exoskeleton was manufactured with fusion deposition modeling using the RAISE3D Pro2 Plus printer using NylonX, a 20% carbon-fiber filled nylon material and PLA. The overall weight of the printed wheelchair-mounted upper limb robotic exoskeleton with hardware is less than 6.61 lb, and it was easily mounted on the back of the powered wheelchair using two screws and did not restrict the movement of the powered wheelchair through regular-sized doors.The printed robotic exoskeleton with motors and sensors mounted to a powered wheelchair is shown in Results Section 3.2.

### 2.2. Surface Electromyography Signal Acquisition and Hand Gestures

The Myo armband was used to acquire surface-level muscle signals known as surface electromyography (sEMG) from the flexor carpi radialis, extensor carpi radialis longus and brevis, palmaris longus, pronator and supinator teres, flexor and extensor carpi ulnaris, brachioradialis, flexor digitorium superficialis, and flexor digitorum communis muscles in the forearm. These muscles generate electrical potentials during finger movements and various hand gestures. Another important decision, besides the features to extract for sEMG signals, was the hand gestures to use for the exoskeleton control. A relaxed open hand [14,15,16,17]; closed hand, or fist, or grasp [14,15,18]; flexion/extension or similar wave in/wave out [15,16,17]; radial and ulnar deviation/flexion [14,15,16,17,18]; pronation/supination [18]; and non-relaxed open or fingers spread [15] are the hand and wrist gestures suggested by various past studies on robotic arm, prosthetic, and exoskeleton control and general EMG signal classification. Five hand gestures were chosen to obtain sEMG muscle signals for classification: wave in, wave out, radial deviation (angled up), ulnar deviation (angled down), and rest. However, 5 gestures alone are not sufficient to control a 3 DOF exoskeleton. Thus, to add to these five gestures without including any other gestures that involve making a fist or articulating the fingers in spread or individual configurations, it was initially decided to include forearm pronation and supination rotations, using a threshold for each direction. Unfortunately, it was found during initial testing that pronation/supination of the forearm was difficult to achieve while the user is strapped into the exoskeleton, so two more hand gestures had to be devised instead. All gestures needed to allow someone to grasp an object, such as a water bottle or the joystick to control a wheelchair. The first five gestures were chosen to perform this task. Other gestures such as fingers spread and a fist, recognized as poses by Thalmic Lab’s software for the Myo armband (Myo Connect/Myo Armband Manager), were avoided due to the conflict of being able to make these poses while trying to grasp an object, as well as the possible confusion by the classification algorithm between the desired movement and the user simply wanting to grasp an object. Thus, the goal was to choose gestures that would support object grasping and other operations without accidentally signaling the movement of the exoskeleton.

**Figure 3:**
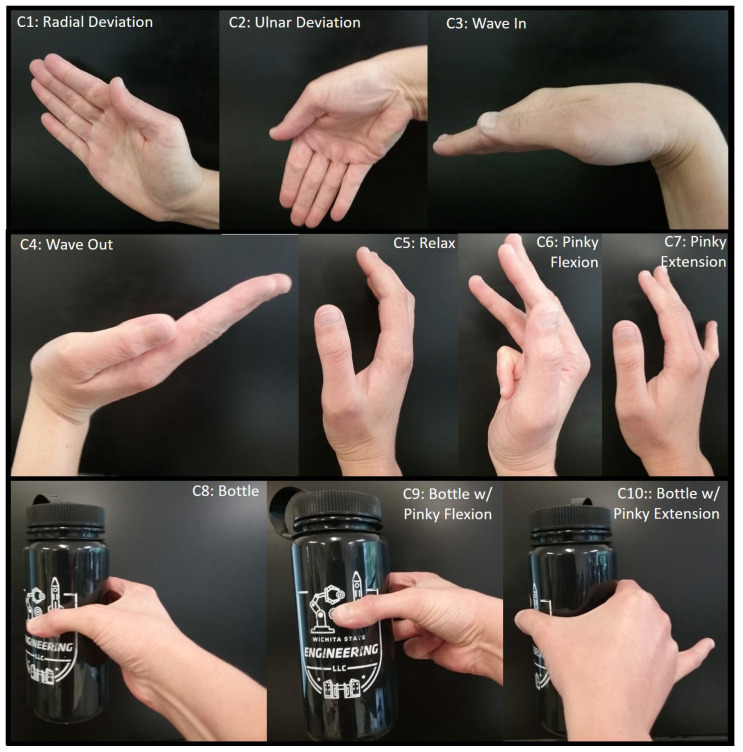
Hand gestures used for exoskeleton control.

A grasp taxonomy of 33 grasp types was identified, with top-level divisions focused on whether the grasp required more power—that is, “all movements of the object have to be evoked by the arm”—or precision, followed by the opposition type or direction of force needed to grasp the object in reference to the palm, namely palm (perpendicular), side (transverse), or pad (parallel) [19]. Interestingly, only for 4 of the 12 precision categories did grasping involve the pinky finger at all, while 2 of the 6 intermediate categories (between precision and power) used the pinky finger. The pinky finger seemed to be more necessary for power grasping, as it appeared to be used in 12 of the 15 grasp schemes shown in the GRASP taxonomy [19]. Hence, it could be concluded that the pinky finger may not be necessary for grasping the majority of the time. Thus, it is proposed that the pinky finger could be flexed and extended while grasping an object, so flexion and extension of the pinky finger were ultimately chosen as the other two hand gestures to control the robotic exoskeleton. This made it possible to have seven different classes of movement in total. Three more redundant classes were also added, making a total of 10 classes, but still with 7 movements, in order to help with pick and place tasks, such as the water bottle pick and place task of this research project. The three additional classes were holding a water bottle as an additional rest position, holding a water bottle while flexing the pinky finger, and holding a water bottle while extending the pinky finger backward. An illustration of the gestures for EMG is shown in Figure 3, with class labels (C1–C10) and gesture names (e.g., radial deviation) labeled on each gesture image. Feature extraction included the use of the RMS, median filter, and mean. Raw sEMG signals were processed through these features, followed by a normalization prior to artificial neural network classification.

Radial deviation, as shown, signals to the exoskeleton controller that the user desires to rotate the elbow joint in the flexion direction, while ulnar deviation signals desired assistance with elbow extension. Waving the hand inward corresponds to shoulder extension, while waving out corresponds to shoulder flexion. Shoulder horizontal adduction is signaled by curling the pinky finger inward towards the palm in a flexion motion without and with the water bottle, while horizontal abduction is signaled by stretching the pinky finger back in extension without and with the water bottle, as shown in the Figure 3. No movement of the exoskeleton is signaled by the relax gesture and the bottle grasping gesture.

### 2.3. Adaptive Controller Mechanism

**Figure 4:**
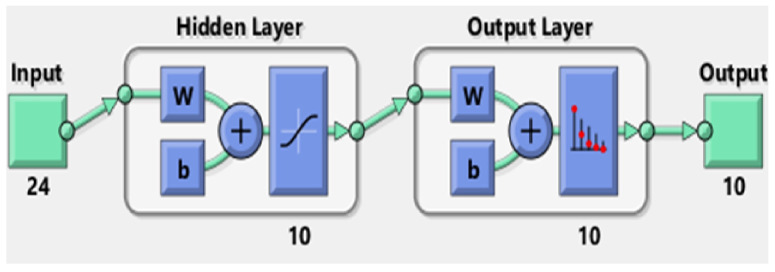
Artificial neural network structure.

MATLAB offers several toolboxes for machine learning, including three for neural networks specifically, namely the Neural Network Pattern Recognition Toolbox, the Neural Fitting app, and the Neural Clustering app. The first two were of interest for this particular study. The Neural Network Pattern Recognition Toolbox only uses scaled conjugate gradient backpropagation for training, while the Fitting app allows a choice between Levenberg–Marquardt, Bayesian regularization, and scaled conjugate gradient backpropagation. Initial testing was conducted to determine which would offer the highest accuracy for these features and number of classes, and it was determined that scaled conjugate gradient backpropagation had the highest accuracy; thus, the scaled conjugate-type neural network was used for all test subjects. The neural network architecture used for the real-time control of the exoskeleton is shown in Figure 4, including the number of input, hidden, and output layers.

The number 24 under the input block represents the 24 features used in this study, and the number 10 under the output block represents the 10 classes; hence, an input vector of length 24 is expected, as is an output vector of length 10. The figure is a basic representation of the 10 hidden layer neural network that was used for testing, with the W block representing an S by R weight matrix, with S referring to the neuron count in that layer and R representing the input vector elements count and the b block representing the bias vector of length S [20].

A trained scaled conjugate backpropagation neural network block with two layers (not to be confused with hidden layers)—one containing netsum and tansig functions and the other containing netsum and softmax functions—was generated. The netsum function is a summing function, while the tansig function is a transfer function, as shown in Equation (1), which calculates the hyperbolic tangent and fits the results between −1 and 1 [21].

(1) $$c=tansig\left(n\right)=2/\left(1+exp{\left(-2*n\right)}^{-1}\right)$$

**Figure 5:**
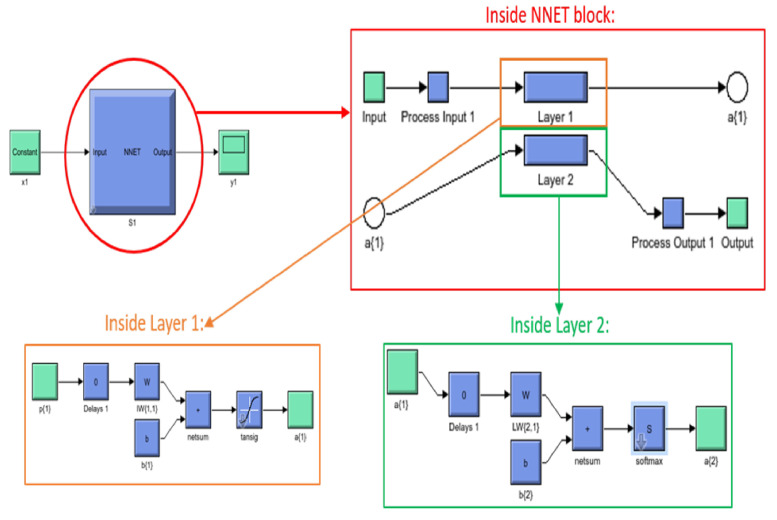
Scaled conjugate backpropagation neural network layers.

The Simulink model for communicating with the Myo armband was supplied by the Myo SDK MATLAB MEX Wrapper [22], a free, open-access package of code and libraries downloadable from GitHub. Initially, the code was built in Simulink only using Arduino encoder reading blocks to read the encoders and Digital Output blocks with stair sequence signal inputs for motor control (as a sort of PWM signal), in addition to the EMG and IMU data acquisition blocks from the Myo SDK MATLAB MEX Wrapper to read the signals from the Myo Armband. This made sense because there was already pre-written Simulink and MATLAB MyoMex code for reading from the armband freely available on GitHub, and the neural pattern recognition toolbox allows for both MATLAB and Simulink. The Simulink blocks making up the tansig and softmax functions are shown in Figure 5.

**Figure 6:**
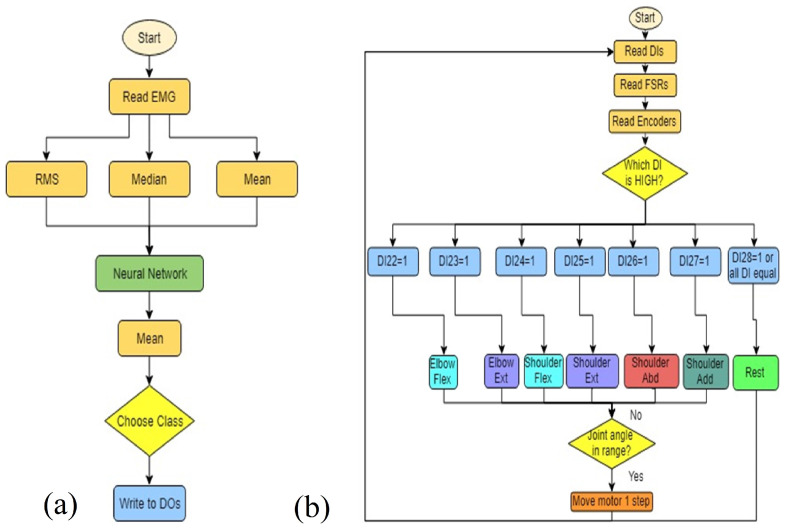
Algorithm flow chart for (**a**) Simulink Myo armband hand gesture recognition and (**b**) Arduino software controller for exoskeleton joints.

While MATLAB scripts could have been utilized for both the Myo armband and neural network, it was quickly discovered that the communication rates between MATLAB and any Arduino board were extremely slow, making MATLAB an impractical option for real-time control. However, it was also found that Simulink alone was not the best option, as when the EMG and IMU blocks were combined with the Arduino encoder blocks and for-loop blocks of stair sequence signals to produce the PWM needed for the stepper motors, this was ultimately too slow for real-time control. Finally, the solution to the timing issue was found by dividing tasks between two Arduino boards and two different software programs, essentially creating parallel computing. Simulink was still used to communicate with the Myo armband, classify gestures with a trained neural network for features of sEMG signals, and to output a total of seven digital values to seven different pins on one Arduino Mega 2560 board. Figure 6b shows a decision making flow chart of the proposed mechanism.

The seven active digital output pins of this first board were then wired to a second Arduino Mega 2560 board. This second Arduino microcontroller was programmed according to our previously published work on a force myography-based exoskeleton controller with some minor changes [23]. The first and most important change, of course, was to read in and use the seven digital inputs from the other board to determine which exoskeleton joint should move and in what direction. FSR values would still also be recorded and displayed, but only as data for post-processing, and perhaps later to add in another layer of safety by using it as an additional logical check. The second revision was to combine all the individual if statements used to allow multiple joints to move at the same time in FSR control into one if statement. This is because it was decided early on that the sEMG control of the exoskeleton would only allow the movement of one exoskeleton joint at the time, with each hand gesture or arm rotation determining the joint and direction of rotation.

## 3. Results

### 3.1. Artificial Neural Network Training and Accuracy

Institutional Review Board (IRB) approval (# 4220) was acquired prior to testing human subjects at Wichita State University. This feasibility study recruited 8 participants between the ages of 18 to 60 years without any upper movement disabilities. Surface EMG signals of participants were recorded and used to train custom neural networks for each test subject. Based on our previous research on hand gesture-based powered wheelchair control and virtual robotic arm control, it was decided to use the scaled conjugate backpropagation type of neural network, due to its higher accuracy and shorter training time [24,25]. The MATLAB neural network pattern recognition toolbox offers several different graphical representations of the accuracy of neural networks, including confusion matrices and error histograms. Confusion matrices show both the count and percentage of false positives, or when the neural network algorithm falsely identifies the class, and true positives. The target class on a confusion matrix plot is the correctly-labeled class, while the output class is the actual labeling of the data by the neural network. A confusion matrix was plotted by the application after the training, validation, and testing stages of the artificial neural network. Test subject 3 had the highest training classification accuracy at 97.6%, while subject 8 had the lowest at 87.3%. For validation accuracies, subject 3 again had the highest at 97.5%, while subject 8 was the lowest at 88.6%. Testing accuracies ranged from 87.6% for subject 8 up to 96.7% for subject 3. For the overall confusion matrices, test subject 3 had the highest accuracy at 97.4%, while subject 8 had the lowest accuracy at 87.7%. For classification accuracy, scores around 85% and upward showed fairly good results for the actual use of the neural network in real-time testing. Therefore, since classification accuracies for testing accuracies of all these eight subjects were over 85%, their neural networks were deemed to be accurate enough to use in the sEMG controller for the exoskeleton. Table 1 shows a summary of the total classification accuracy of each subject’s neural network during each phase of the neural network training.

### 3.2. Human Subject Testing Results

**Figure 7:**
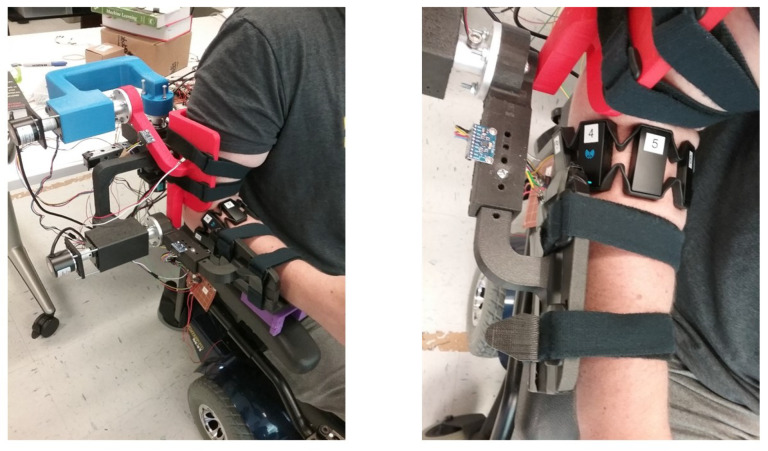
Human subject testing setup views (isometric and top).

Each participant was given two trials to individually control each joint of the exoskeleton using the proposed hand gestures followed by a water bottle pick and place task. The exoskeleton setup is shown in Figure 7, where a participant was seated on a powered wheelchair and wore the actively-actuated exoskeleton with a Myo armband on the right forearm. Each participant’s individual joint movements (Section 3.2.1) and water bottle pick and place (Section 3.2.2) tasks were recorded.

#### 3.2.1. Individual Joint Movements

**Figure 8:**
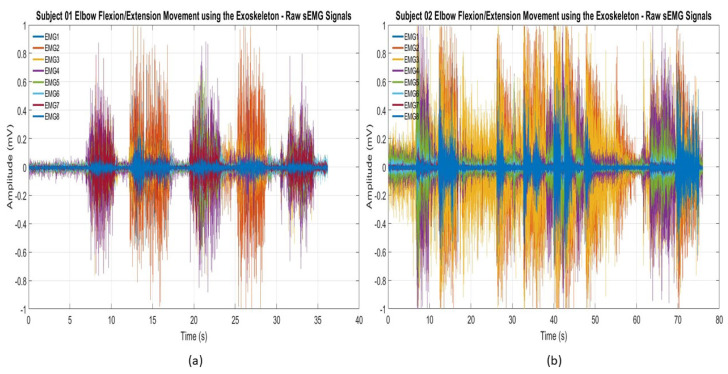
Raw sEMG signals recorded during the elbow flexion/extension movements of the exoskeleton: (**a**) subject 01 and (**b**) subject 02.

To evaluate the overall efficacy of the control system in real time, all the participants data sets were recorded during the experimental testing of the exoskeleton. Median and mean values of different movement types for each participant were calculated and represented in box plots. Each participant was given two trials to control individual joints of the exoskeleton, and their second trial was recorded. The adaptive control mechanism limits any joint movement beyond 70°, which ranges from +35° to −35° at the rest condition. This plays an important role in human safety because it can override human intent and ensure the user’s safety. Figure 8 shows the raw electromyography signals from two participants’ forearms while controlling the exoskeleton’s individual joints using hand gestures. These raw sEMG signals show distinct patterns thats clearly explain the need for mathematical features to be determined by an artificial neural network to accurately identify hand gestures. A similar strategy can also be applied in future research to test people with upper-arm muscle weakness.

**Figure 9:**
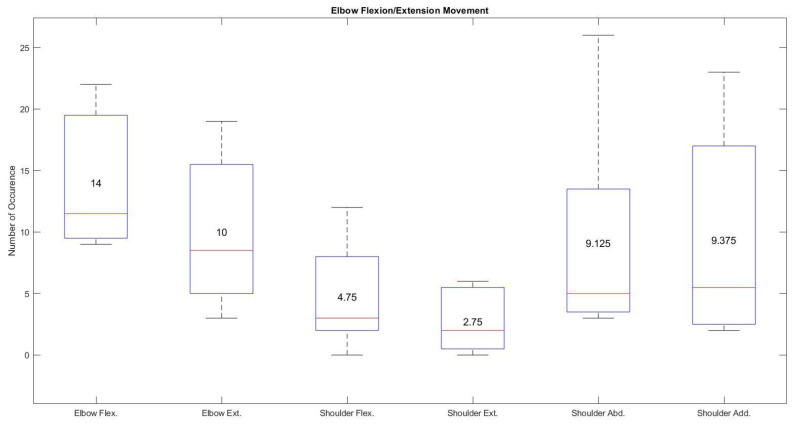
Average movement occurrence of all the joints during the specific elbow flexion/extension movement using hand gestures.

Each participant’s trained artificial neural network was implemented into the controller unit to identify the number of occurrences for each joint while controlling each individual joint at a time, mimicking the natural movement of the arm. For example, if a user only used specific gestures to control elbow flexion/extension movement, there is a possibility that the user could perform other gestures associated with shoulder flexion/extension or shoulder abduction/adduction movements. Figure 9 demonstrates the number of occurrences of all three exoskeleton joints during the elbow flexion/extension movement. The highest median and mean values recorded for elbow flexion and extension were 11.5 and 14 and 8.5 and 10, respectively. These values are higher than the median and mean values of shoulder flexion (3 and 4.75) and extension (2 and 2.75) and shoulder horizontal abduction (5 and 9.125) and adduction (5.5 and 9.375) readings. This type of adaptability not only improves user acceptance but also allows more than one degree of freedom to mimic the natural movement of the arm. Average elbow flexion was recorded at 30%, while average elbow extension was 20%, yielding a combined total of 50% for the elbow flexion or extension movements.

**Figure 10:**
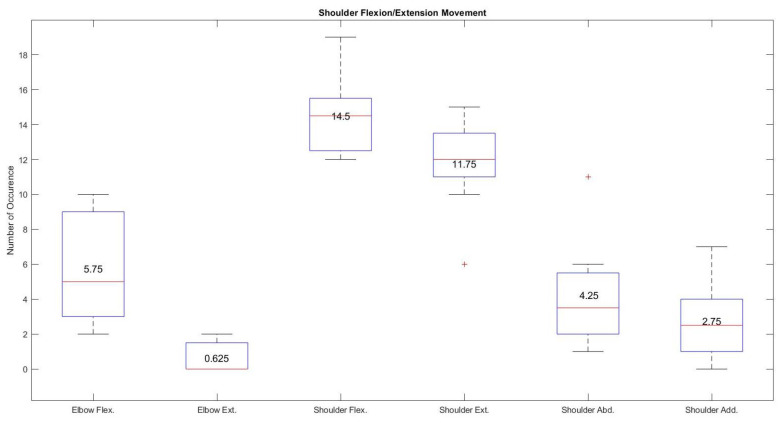
Average occurrence of all the joints during the specific shoulder flexion/extension movement using hand gestures.

The shoulder flexion/extension movement of the exoskeleton using hand gestures seems to exhibit the best performance based on the recordings from all the participants. Figure 10 shows high median and mean values of 14.5 and 14.5 for shoulder flexion and 12 and 11.75 for shoulder extension respectively compared with the very low median and mean values recorded from elbow flexion (5.75 and 8), elbow extension ( 0 and 0.625), shoulder abduction (3.5 and 4.25), and shoulder adduction (2.5 and 2.75). These results show that the hand gestures (wave in and wave out) associated with this task made it very easy to control the specific joint compared with other elbow flexion/extension or shoulder horizontal abduction/adduction movements.

**Figure 11:**
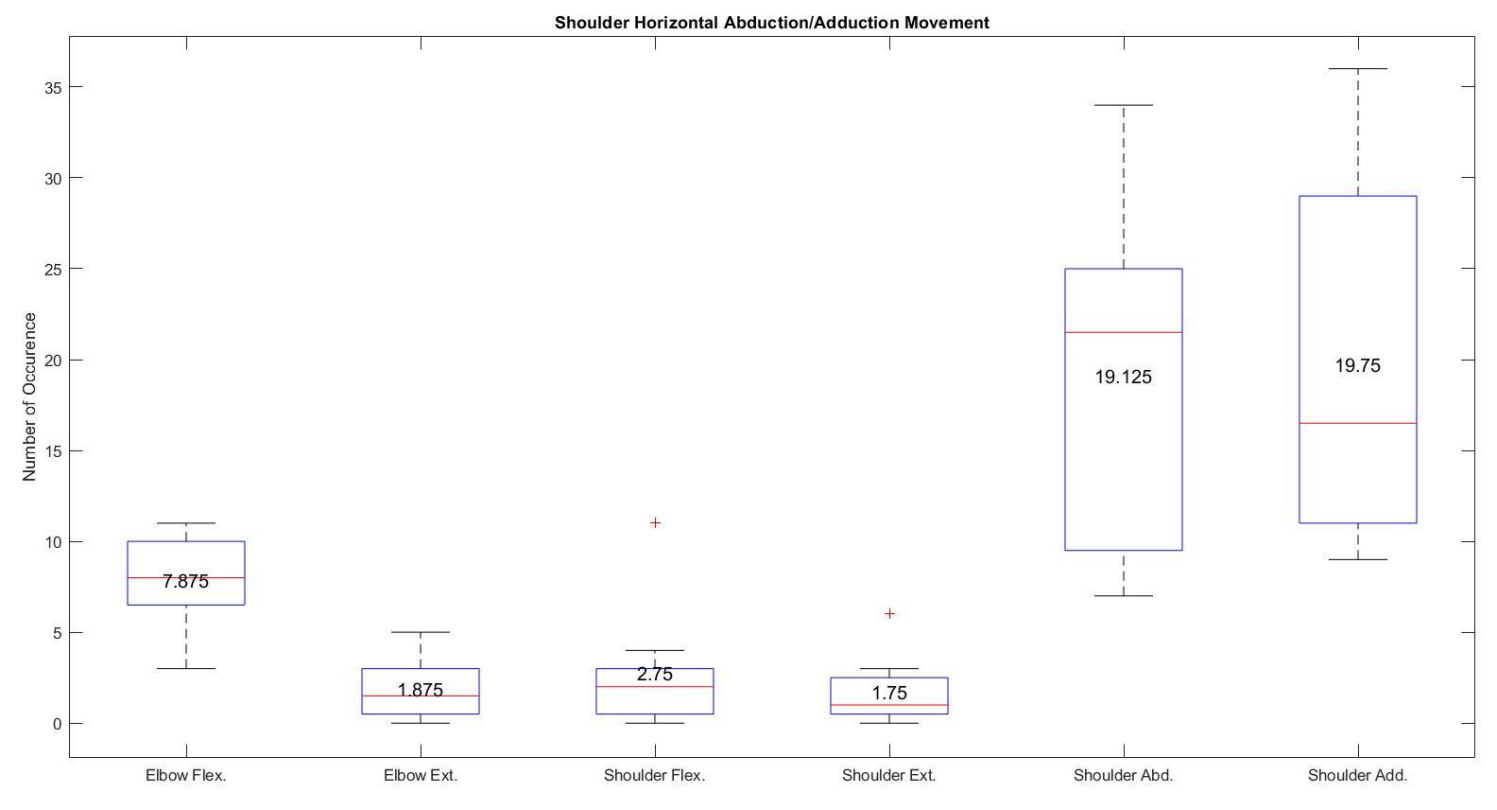
Average occurrence of all the joints during the specific shoulder horizontal abduction/adduction movement using hand gestures.

Shoulder horizontal abduction/adduction movement of the exoskeleton was often mistaken by the neural network during the individual testing of the joint. Figure 11 shows higher occurrences in terms of median and mean values (21.5 and 19.125 for shoulder horizontal abduction and 16.5 and 19.75 shoulder horizontal adduction) during the shoulder horizontal abduction/adduction movement using the associated gestures (pinky finger flexion/extension). Despite having a higher occurrence rate using pinky finger movements for shoulder horizontal abduction and adduction movements, quick changes in hand gesture classification were observed where elbow flexion/extension movement falsely identified by the trained artificial neural network due to the use of the pinky finger in radial and ulnar deviations.

#### 3.2.2. Water Bottle Pick and Place Task

All eight participants who completed the individual joint tests with the EMG controller also successfully completed the water bottle pick and place task. The shortest time for completing the water bottle pick and place task was 15.3 s by subject 5, while the longest time was 62.6 s by subject 3. A summary of the times for the hand gesture-based adaptive controller used in the water bottle pick and place task is shown in Table 2. The average completion time of the water bottle pick and place task for EMG control was 36.35 s.

**Figure 12:**
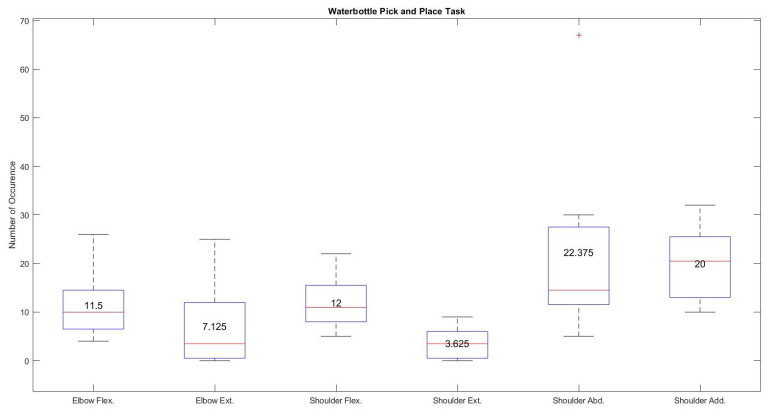
Number of occurrences during water bottle pick and place task using hand gestures.

Figure 12 shows the number of occurrences in terms of median and mean values for each joint of the exoskeleton during a water bottle pick and place task. The median and mean values of shoulder horizontal abduction and adduction were 14.5 and 22.375 and 20.5 and 20, respectively. These values, compared with scores of 10 and 11.5 for elbow flexion, 3.5 and 7.125 for elbow extension, 11 and 12 for shoulder flexion, and 3.5 and 3.625 for shoulder extension, demonstrates the higher use of shoulder horizontal abduction and adduction during a water bottle pick and place task compared with the almost equally utilized elbow and shoulder flexion and extension movements.

The most used movements for the water bottle pick and place task with the proposed controller were, on average, shoulder adduction at 30% and abduction at 28%, while shoulder flexion was used 20% of the time on average and elbow flexion was used 15% of the time, followed by elbow extension at 7% and shoulder extension at only 5% of the time. The high use of shoulder adduction/adduction could be misleading if taken purely as intention. Visual observations of the tests showed that desired shoulder abduction and adduction movements were often confused with each other, which was also evident in the individual shoulder abduction/adduction joint test. A summary of these average joint movement occurrences for all eight participants, with the intended movements highlighted, is given in Table 3. The highlighted results prove that each participant’s specific hand gesture associated with specific joint control of the exoskeleton was recognized by the trained artificial neural network followed by generating control signals for that specific joint. For example, first three columns of the Table 3 identify the maximum and minimum usage of the exoskeleton joint which clearly shows that the adaptive controller was able to maneuver a specific joint during the specific task. The fourth column of the Table 3 shows majority of the participants used shoulder horizontal abduction and adduction to perform water bottle pick and place compared with shoulder and elbow flexion and extension movements.

Table 4 shows subjective feedback on the ease of the proposed hand gestured-based controller for individual joint control. The average results are on a rating of 1–5, with 5 showing that the controller was easy to control and 1 representing that it was difficult for questions listed in the Table 4. The scores from all eight participants were averaged together. Shoulder inward/flexion movement received the highest preference, whereas the rotating inward shoulder joint movement was noted as being least preferred.

## 4. Discussion

Experimental testing on human subjects without upper arm movement disability showed the need for improvements in the upper arm flexion/extension assembly angle for better fitting, comfort, and classification and improvements in registering hand gestures for classification.

### 4.1. Fitting and Comfort

Despite having a compact design of the exoskeleton, participants with a smaller upper arm radius felt a greater gap between the posterior side of the upper arm and the upper arm assembly. Due to this gap, participants with a small arm radius sometimes felt a minor slippage of the upper arm, specifically during shoulder flexion movement. This gap was filled with a plastic foam to restrict the upper arm from slipping during exoskeleton control. The shoulder’s complex anatomy makes it very difficult to design an exoskeleton that mimics the rotation axis of the exoskeleton joint that can align with the human joint and offer all degrees of freedom when the exoskeleton robot is attached to the exterior of the human body [26,27]. Future designs of the prototype can include an angular joint assembly of the upper arm exoskeleton that can reduce the gap between the exoskeleton and upper arm assembly. A future design will include a locking hinge mechanism for the upper arm exoshell to adjust to the user’s shoulder angle along the anatomical frontal plane, as well as the isolation of the links between shells and motor cases from the surfaces interfacing directly with the user. The interfacing surfaces can probably still continue to be fusion deposition modeling-printed PLA or NylonX materials, as well as the motor cases; however, the separated links will need to be manufactured out of a stronger material, such as aluminum. The exoshells in the future design will be made more rounded, maybe using the 3D scanning of an arm to gather further information about the geometry of the arm, although the current open square design allows for a larger range of arm sizes. One of the participants commented that flexion of the elbow with the Myo armband on the forearm could cause pinching because of its size and shape.This issue restricted wrist movements and was observed during the experimental system setup with one participant. The use of flexible sEMG sensors has the potential to reduce the pinching effect.

### 4.2. Hand Gestures and Classification

The use of a feed forward artificial neural network with 10 hidden layers achieved the highest overall accuracy of 97.4% and the lowest accuracy of 87.7%. Despite its very high accuracy, the overall system requires a computer with Matlab^®^ software installed in order to implement the trained artificial neural network. The use of a Simulink-based model slows down the real-time control operation due to its limited data transfer rate. This issue also relates to the lower sampling rate of the Myo armband (200 Hz). The future work can utilize an on-board processor with a built-in trained network and an EMG system with a faster sampling rate to minimize delays in feature extraction and translation. Two out of eight participants reported issues regarding pinky finger gestures for the shoulder joint actuation of the exoskeleton while holding an object in their hand. This issue limits participants providing a higher gripping force, which will be important for lifting heavy objects in the near future. With regards to control, the data, survey results, and general observations show that the shoulder abduction and adduction control with the proposed hand gestures need to be improved, due to difficulties in signaling user intent to begin with and misclassification in recognition patterns from sEMG signals from the human forearm.

## 5. Conclusions

This paper successfully demonstrates an integration of artificial neural network-based hand gesture recognition with an adaptive upper limb exoskeleton device to extract 10 gestures in order to actuate a 3 DOF wheelchair-mounted upper limb robotic exoskeleton for activities of daily living. Overall, the trained scaled conjugate gradient neural network achieved an average of more than 93% accuracy using root mean square, medial filter, and mean features. The average movement occurrence shows a higher acceptance of the proposed technology for shoulder flexion/extension followed by elbow flexion/extension and shoulder horizontal abduction/adduction movements. The designed exoskeleton was printed using PLA and NylonX, weighs less than 6.61 lb, and can be easily mounted on the back of a powered wheelchair. All the participants were easily able to adapt to the proposed controller mechanism without any prior experience and performed individual joint movements followed by a water bottle pick and place task. The fastest response recorded while performing the water bottle pick and place task using the proposed technology was 15.3 s, which can be compared with the longest response (62.6 s), demonstrating the variability in user acceptance rates. Subjective feedback from each participant shows a higher acceptance rate (an average score of 4.125 out of 5) for bending the exoskeleton shoulder joint, whereas the lowest average score of 2.875 out of 5.0 was found for rotating the exoskeleton shoulder joint towards the body (shoulder horizontal adduction). Future work will include testing a force myography (FMG)-based controller mechanism and a comparison between EMG and FMG controller schemes based on real-time control and subjective feedback from participants, which will be beneficial for future work on people with muscular dystrophy.

## Figures and Tables

**Table 1:** Artificial neural network classification accuracy.

Subject #	Training Accuracy %	Validation Accuracy %	Testing Accuracy %	Overall Accuracy %
1	92.3	91.5	91.1	92.0
2	90.2	90.4	89.9	90.2
3	97.6	97.5	96.7	97.4
4	96.4	96.2	95.7	96.3
5	90.9	90.0	90.1	90.8
6	89.3	87.5	89.0	89.1
7	89.9	91.1	89.9	89.9
8	87.8	88.6	87.6	87.7

**Table 2:** Water bottle pick and place time using the proposed hand gestured-based artificial neural network-trained adaptive wheelchair-mounted upper limb robotic exoskeleton.

Subject #	Completion Time (sec)
1	27.7
2	21.2
3	62.6
4	51.2
5	15.3
6	39.4
7	24.2
8	49.2

**Table 3:** Average joint movement occurrence during individual joint control and water bottle pick and place task using the wheelchair-mounted upper limb robotic exoskeleton.

Motion/Task	Elbow Flex/Ext %	Shoulder Flex/Ext %	Shoulder Horizontal Abd/Add %	Water Bottle Pick & Place %
Elbow Flex	30	14	16	15
Elbow Ext	20	2	3	7
Shoulder Flex	10	37	5	20
Shoulder Ext	5	31	3	5
Shoulder Horizontal Abd	17	10	35	28
Shoulder Horizontal Add	16	6	37	30

**Table 4:** Subjective feedback on exoskeleton joint control.

Subjective Feedback Questions	Average Score (0–5)
Bending exoskeleton elbow joint inward	3.75
Straightening out exoskeleton elbow joint	3.25
Bending exoskeleton shoulder joint inward	4.125
Straightening out exoskeleton shoulder joint	3.375
Rotating inward (towards body) exoskeleton shoulder joint	2.875
Rotating out (away from body) exoskeleton shoulder joint	3.125
